# Bone chips, fibrin glue, and osteogeneration following lateral suboccipital craniectomy: a case report

**DOI:** 10.1186/1756-0500-6-523

**Published:** 2013-12-09

**Authors:** Thomas Linsenmann, Camelia M Monoranu, Almuth F Kessler, Ralf I Ernestus, Thomas Westermaier

**Affiliations:** 1 Departments of Neurosurgery, University of Würzburg, Josef-Schneider-Str. 11, Würzburg D-97080, Germany; 2 Departments of Neuropathology, University of Würzburg, Josef-Schneider-Str. 11, Würzburg D-97080, Germany

**Keywords:** Bone chips, Fibrin glue, Osteogeneration, Lateral suboccipital craniectomy

## Abstract

**Background:**

Suboccipital craniectomy is a conventional approach for exploring cerebellopontine angle lesions. A variety of techniques have been successfully employed to reconstruct a craniectomy. This is the first report about the histological findings after performing a cranioplasty by using a mixture of autologous bone chips and human allogenic fibrin glue.

**Case presentation:**

A 53-year-old German woman underwent left lateral suboccipital retrosigmoidal craniectomy for treatment of trigeminal neuralgia in 2008. Cranioplasty was perfomed by using a mixture of autologous bone chips and human allogenic fibrin glue. Due to recurrent neuralgia, a second left lateral suboccipital craniectomy was performed in 2012. The intraoperative findings revealed a complete ossification of the former craniotomy including widely mature trabecular bone tissue in the histological examination.

**Conclusion:**

A mixture of autologous bone chips and human allogenic fibrin glue seems to provide sufficient bone-regeneration revealed by histological and neuroradiological examinations.

## Background

The retrosigmoid approach via a suboccipital craniectomy is a conventional approach for exploring cerebellopontine angle lesions. This approach is considered the simplest route to the cerebellopontine angle and lateral clivus. This route may be used in a variety of surgeries, such as tumor removal, vestibular neurectomy, brainstem auditory implantation and neurovascular decompression. A variety of techniques have been successfully employed to reconstruct craniectomy and/or craniotomy bone gaps such as bone grafts, silastic, acrylic or metal plates, hydroxyapatite cement or ceramic implants.

Cranioplasty using a mixture of autologous bone chips and human allogenic fibrin glue is a well known technique first reported in 1997 by Sawamura *et al*. We report a case of a patient using this technique in 2008 with a surprising intraoperative and histological result concerning the morphology while performing a second operation in the same patient in 2012.

## Case presentation

**Figure 1:**
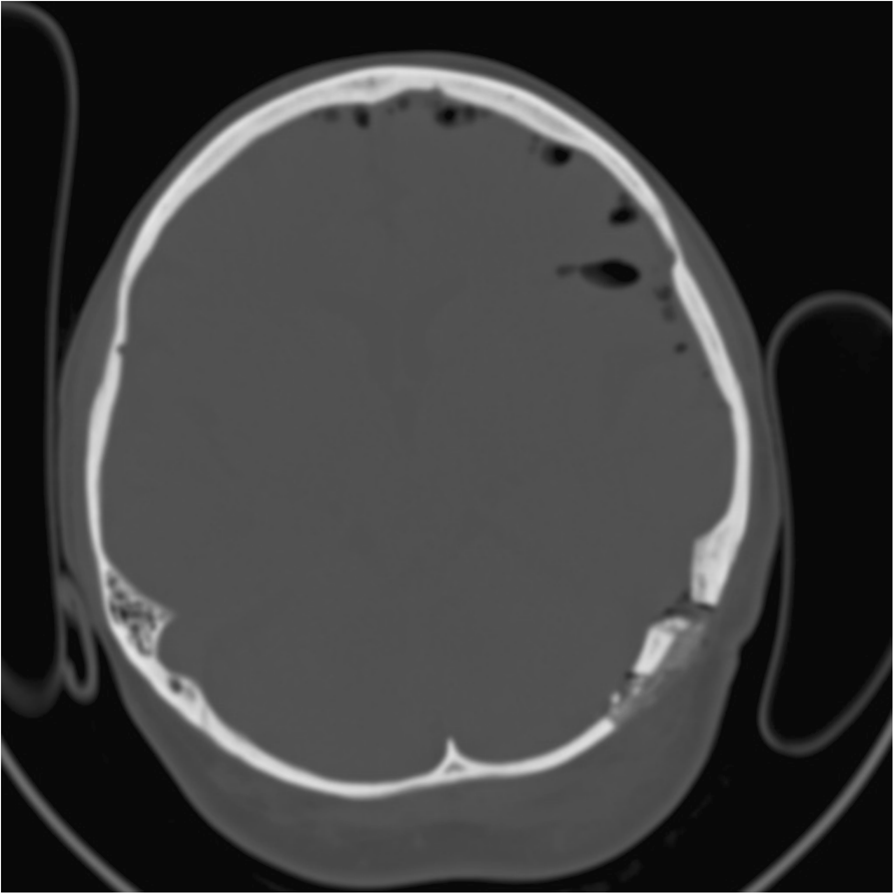
**Axial bone-window computed tomography scan performed one day after first operation.** The mass of bone chips and fibrin glue can be well identified.

**Figure 2:**
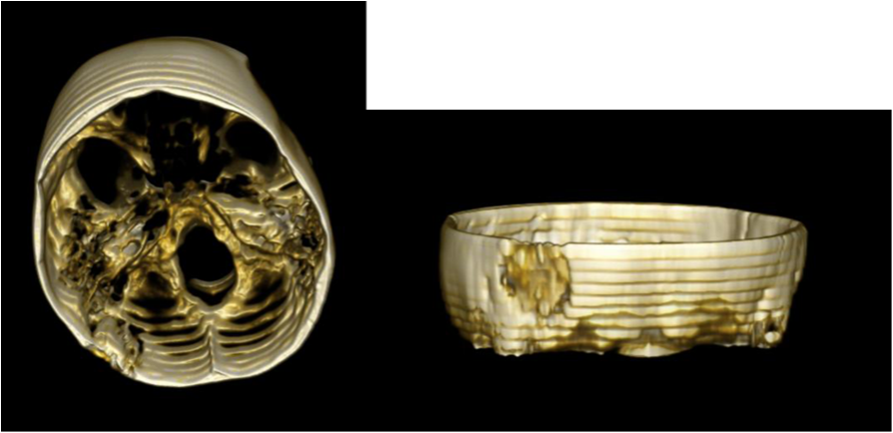
**Three-dimensional computed tomography scan one day after first operation.** Bone chips sealed in fibrin glue in the left retromastoid occipital bone.

A 53-year-old woman underwent left lateral suboccipital retrosigmoidal craniectomy for treatment of trigeminal neuralgia in 2008. After water tight suture of the dura mater, crushed bone fragments were sprayed with a fibrinogen and thrombin mixture according to the pharmaceutical and technical instructions of this device (Tissucol Duo 1 ml Immuno-Solution®, Baxter, Germany). Then the admixture was placed on the dura mater and shaped to fit in the bone defect followed by replacing the muscle flap. Further postoperative course was uneventful. Postoperative computed tomography (CT) scan one day after surgery revealed correct placement of the mass of bone chips sealed in fibrin glue [Figures 1 and 2].

**Figure 3:**
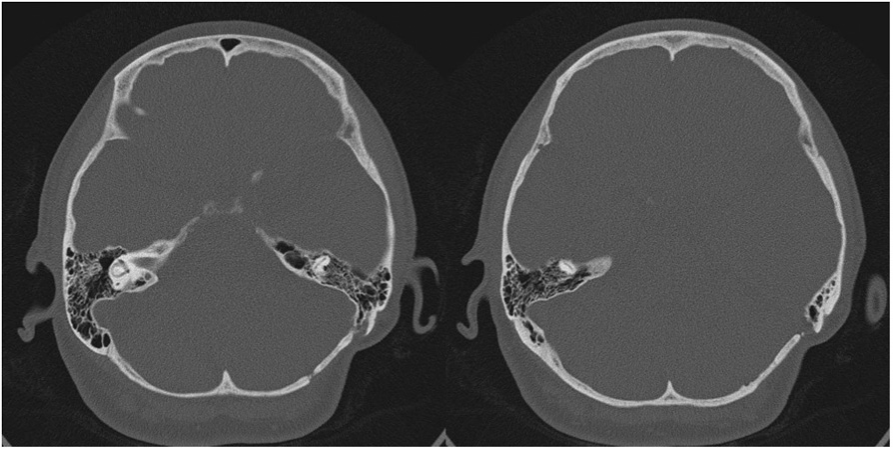
Preoperative bone-window computed tomography scan 4 years after first operation demonstrates nearly complete ossification.

**Figure 4:**
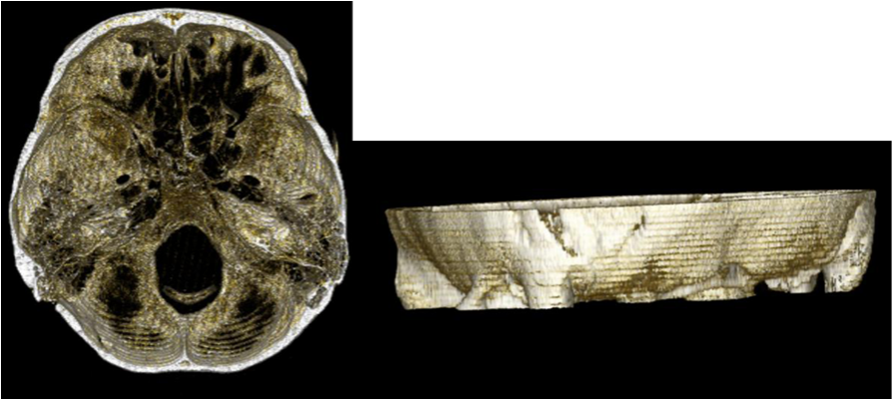
Three-dimensional computed tomography scan 4 years after first operation also demonstrates a complete smooth bone plate.

Due to recurrent neuralgia, a second left lateral suboccipital craniectomy was performed in 2012. The preoperative axial-bone-window CT scan revealed that the bone was in the top level [Figure 3]. Three-dimensional CT also demonstrated a complete smooth bone plate (Figure 4).

**Figure 5:**
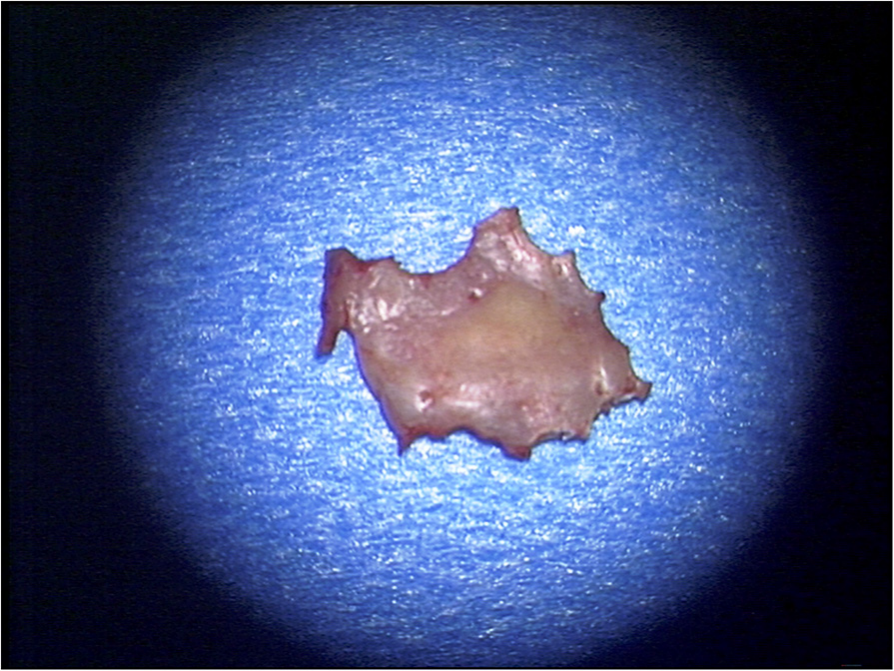
Cranioplasty after performing a new far lateral suboccipital retrosigmoidal approach.

**Figure 6:**
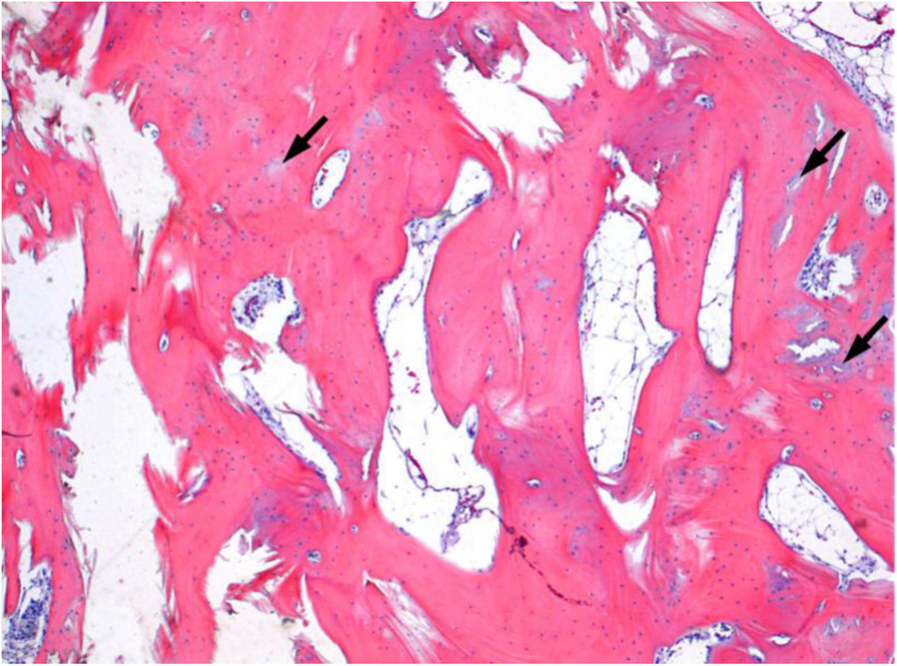
Trabecular bone tissue, predominantly mature, with only small areas of incomplete mineralization (osteoid).

The intraoperative findings revealed a complete ossification of the former craniotomy. There was no clear border between the original bone and the cranioplasty. After performing the new far lateral suboccipital retrosigmoidal approach via a new burr hole, the cranioplasty was preserved for further histological examination (Figure 5), which confirmed a widely mature trabecular bone tissue with several circumscribed areas of osteoid (incompletely mineralized tissue) (Figure 6). Afterwards, the new cranioplasty was performed according to the technical steps in the first operation.

## Discussion

In contrast to Sawamura *et al.*[1], we could investigate intraoperatively the primarily performed cranioplasty due to a recurrent neuralgia of this patient. Thus further histological examinations were possible.

The physiology of osteoregeneration mediated by fibrin is unknown. Fibrin glue itself has no osteo-inductive power [2]. Neither biochemical nor histological parameters revealed a direct influence of fibrin sealant on any osteoinductive process [3].

Using biphasic calcium phosphate as an osteoconductive scaffold in combination with fibrin glue is already well described as an alternative to autogenous bone graft or methyl methacrylate for posttraumatic calvarium bone defect reconstruction [4].

As a cell-delivery vehicle, fibrin glues seems to facilitate cell attachment, growth and differentiation and, ultimately, tissue formation and organization by its three-dimensional structure [5].

## Conclusions

The present case demonstrates that a stand-alone implantation of a fibrinogen and thrombin mixture seems to provide sufficient bone-regeneration revealed by histological and neuroradiological examinations.

It provides an easy way of sufficient rebuilding of new bone after performing a craniectomy in the posterior fossa. Further studies should reveal whether this technique is useful after craniectomy in case of other indications, e.g. tumor resections with following radio-chemotherapy.

The well described capacity of cell attachment because of its structure may indicate further studies to mix stem cells or growth factors with fibrinogen and thrombin glue to research the ability of building new sufficient bone.

## Consent

Written informed consent was obtained from the patient for publication of this case report and accompanying images. A copy of the written consent is available for review by the Editor-in-Chief of this journal.

## Abbreviations

CT: Computed tomography.

## Competing interests

All authors declare that they have no competing interests.

## Authors’ contributions

TL collected data for the case report and drafted the manuscript. CMM performed the histological examinations. AFK and RIE critically revised the manuscript. TW was involved in drafting the manuscript and gave final approval for publication. All authors read and approved the final manuscript.
